# Comparison of the morphological characteristics of the choroidal sublayer between idiopathic macular holes and epiretinal membranes with automatic analysis

**DOI:** 10.1186/s12886-023-03027-8

**Published:** 2023-06-16

**Authors:** Shijie Zhang, Junmeng Li, Wenbo Zhang, Yanzhen Zhang, Xiaopeng Gu, Yadi Zhang

**Affiliations:** Department of Ophthalmology, Peking University First Hospital, No. 8 Xi Shi Ku Street, Xicheng District, Beijing, 100034 China

**Keywords:** Idiopathic macular hole, Idiopathic epiretinal membrane, Optical coherence tomography (OCT), Choroidal sublayers, Choroidal vascular index (CVI), Automated segmentation

## Abstract

**Purpose:**

To compare the choroidal sublayer morphologic features between idiopathic macular hole (IMH) and idiopathic epiretinal membrane (iERM) on spectral-domain optical coherent tomography (SD-OCT) using an automatic segmentation model.

**Methods:**

Thirty-three patients with idiopathic IMHs and 44 with iERMs who underwent vitrectomies were involved. The enhanced depth imaging mode of SD-OCT was used to obtain the B-scan image after single line scanning of the macular fovea. The choroidal sublayer automatic analysis model divides the choroidal into the choroidal large vessel layer, the middle vessel layer and the small vessel layer (LVCL, MVCL and SVCL, respectively) and calculates the choroidal thickness (overall, LVCL, MVCL and SVCL) and vascular index (overall, LVCL, MVCL and SVCL). The morphological characteristics of the choroidal sublayer in the ERM eyes and the IMH eyes were compared.

**Results:**

The mean choroidal thickness in the macular centre of the IMH eyes was significantly thinner than that of the ERM eyes (206.35 ± 81.72 vs. 273.33 ± 82.31 μm; *P* < 0.001). The analysis of the choroidal sublayer showed that the MVCL and SVCL macular centres and 0.5–1.5 mm of the nasal and temporal macula were significantly thinner in the IMH eyes than in the ERM eyes (*P* < 0.05), and there was a difference in the macular centre of the LVCL between the two groups (*P* < 0.05). In contrast, the choroidal vascular index of the macular centre in the IMH eyes was significantly higher than that in iERM eyes (0.2480 ± 0.0536 vs. 0.2120 ± 0.0616; *P* < 0.05). There was no significant difference in the CVI of other parts of the macula, the LVCL or MVCL between the two groups.

**Conclusion:**

The choroidal thickness of the IMH eyes was significantly thinner than that of the iERM eyes, which was mainly observed in 3 mm of the macular centre and the MVCL and SVCL layers of the choroid. The choroidal vascular index of the IMH eyes was higher than that of the iERM eyes. These findings suggest that the choroid may be involved in the pathogenesis of IMH and iERM.

## Key messages

1. The pathogeneses of idiopathic macular hole (IMH) and idiopathic epiretinal membrane (iERM) are closely related to the relationship between the vitreous and the macula. Changes in the choroid are likely to be related to local pathological changes in the macula.

2. The choroidal thickness of the IMH eyes was significantly thinner than that of the iERM eyes, which was mainly observed in 3 mm of the macular centre and the middle and small vessel layers of the choroid.

3. The choroidal vascular index of the IMH eyes was higher than that of the iERM eyes. These findings suggest that the choroid may be involved in the pathogenesis of IMH and iERM.

## Introduction

An idiopathic macular hole (IMH) and idiopathic epiretinal membrane (iERM) are common manifestations in the macula in elderly individuals. IMHs are retinal defects in the fovea [1], and approximately two-thirds of affected patients are women [2]. It has been reported that the incidence rate of IMH in the general population is between 0.2% and 0.8% [3, 4]. An idiopathic epiretinal membrane is a fibrocellular proliferation at the vitreoretinal interface on the inner retinal surface, which leads to visual impairment or metamorphopsia. The prevalence of iERM did not differ significantly between men and women [5–7]. Population studies have shown that the overall iERM prevalence ranges from 7% to 11.8%, while the 5-year incidence is 5.3% [8–10]. An IMH and the iERM have similar appearances when they occur in the macular region; both are related to age, especially in elderly individuals. There was no pathological process of neovascularization in both diseases. The key point is that the pathogenesis of both is closely related to the relationship between the vitreous and the macula.

The possible pathogenesis of an IMH is related to the abnormal adhesion of the vitreous and macula. The posterior vitreous cortex applies direct anterior and posterior traction to the macular fovea [1, 11, 12]. An abnormal vitreous macular adhesion produces dynamic traction, and the longitudinal contraction of collagen fibres leads to progressive forward traction until a hole develops [13–15]. Posterior vitreous detachment (PVD) plays a critical role in the pathogenesis of the iERM [5, 16, 17]. The residual vitreous remnants on the retinal surface after PVD development, including hyalocytes, provide a medium for proliferation and transdifferentiation of glial cells. These hyalocytes stimulate Müller cells to send the process through an intact inner limiting membrane (ILM) to form a scaffold. Activated Müller glial cells lead to the formation of the preretinal membrane [18].

The macular fovea is a nonvascular area, and 100% of its blood supply comes from the choroid. Therefore, macular disease may affect choroidal blood flow and thus choroidal thickness. Similarly, changes in choroidal blood flow and thickness may also affect macular disease. The difference in choroidal function between IMH eyes and iERM eyes may be related to the different clinical manifestations of the two macular diseases. At present, choroidal thickness and blood flow is measured in vivo by OCT. The measured parameters include choroidal thickness and choroidal vascular index (CVI). Enhanced depth imaging (EDI-OCT) has allowed more accurate evaluation of choroidal thickness, provides detailed information about the choroid, and makes highly reliable and repeatable measurements of choroidal thickness [19, 20]. In addition, the CVI is another choroidal vascular marker based on EDI-OCT. It is calculated as the ratio of the vascular area to the matrix area by the image binarization method [21]. It is considered to be a more reliable parameter than choroidal thickness [22]. In contrast to choroidal thickness, it has been reported that the CVI is not affected by many biological variables, such as intraocular pressure, axial length, diurnal variation, and refractive error [23].

Many studies have observed the relationship between an IMH, the iERM and choroidal thickness. EDI-OCT has been used to compare the choroidal thickness [24–26] between IMH patients and control groups. The results showed that the choroid became thinner in the patients with IMHs, suggesting that the decrease in choroidal blood flow may be related to the presence of an IMH. The relationship between the choroid and the iERM is unclear. Previous studies have observed choroidal thinning in newly developed or significantly progressed iERM eyes and choroidal thickening in spontaneously resolved iERM eyes [27]. Several studies have found that choroidal thickness decreases after vitrectomy and that the iERM peels [28, 29]. Studies have found that the choroidal thickness of the iERM with contraction is significantly thickened [30]. Other studies found that there was no significant change in the CVI in iERM patients [31]. These studies show that the choroid is involved in the occurrence and development of an IMH and the iERM.

Due to the similarity between an IMH and the iERM and their relationship with the choroid, the comparison of the choroidal structure between an IMH and the iERM is helpful to understand the occurrence and development of an IMH and the iERM. To the best of our knowledge, there is no comparison of choroidal structures between an IMH and the iERM. In this study, we conducted a retrospective study using a new choroidal sublayer segmentation model based on deep learning to compare the choroidal sublayer thickness and CVI of an IMH and the iERM to observe the different morphological characteristics of the foveal choroidal sublayer in the two macular diseases and the possible relationship between the two diseases.

### Subjects and methods

We retrospectively analysed the medical records of patients who underwent vitrectomy for an IMH and an iERM at the Ophthalmology Department of Peking University First Hospital between 1 January **2017**, and 30 June 30 2021. The inclusion criteria were as follows: 1) IMH and iERM were diagnosed by OCT and treated with vitrectomy; 2) clear ocular media; and 3) a high-quality image was acquired to precisely measure choroidal thickness. The exclusion criteria were as follows: eyes with myopia of more than 6 dioptres; ocular trauma or tumour; eyes with other ocular pathologic features that could have interfered with functional results (such as glaucoma, previous uveitis, visually significant cataract or age-related macular degeneration); history of intravitreal drug injection, retinal surgery or refractive surgery; history of systemic corticosteroid use; or any systemic disease affecting the eyes. The Institutional Review Board waived the need for patient consent for this retrospective study (Peking University First Hospital No:2022–312).

All patients underwent a comprehensive ophthalmic assessment, including best corrected visual acuity, slit lamp biomicroscopy, intraocular pressure (IOP) measurement, fundus examination, refractive error examination (rm8900; TOPCON) and axial length measurement (Carl Zeiss Meditec AG, Jena, Germany). Snellen visual acuities were converted to the logarithm of the minimal angle of resolution (log-MAR) for statistical analysis.

### Measurement of choroidal thickness and CVI

OCT images of IMH and iERM before surgery were used for analysis. The EDI mode of SD-OCT (Heidelberg Engineering, Heidelberg, Germany) was used to obtain the B-scan image after single-line scanning of macular fovea. A single B-scan consists of 768 A-scans (high-speed mode). To improve the signal-to-noise ratio, automatic real-time tracking is adopted. The B-scan images of single line scanning are composed of 100 two-dimensional images, which can improve the clarity of the image and remove the image artefacts. The OCT B-scan image set needs to have sufficient quality to analyse the choroid, and the quality of the images included in the study is greater than 28. The diagnosis and staging of IMH were determined according to the classification by Gass [1]. The ERM was classified into four stages by spectral-domain optical coherence tomography as previously described by Govetto et al. [32].

**Fig. 1:**
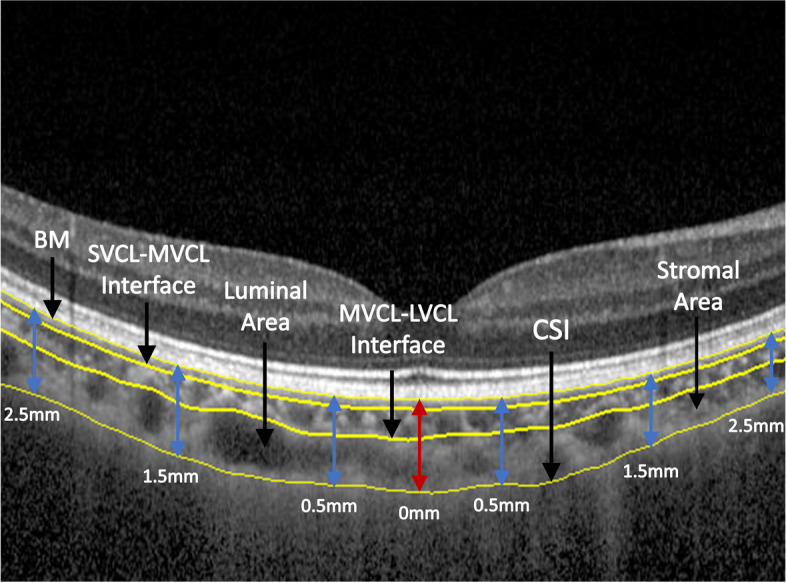
Measurement of choroidal thickness and CVI

The cross-sectional structure of the choroid can be displayed on EDI-OCT B-scan images. To study the morphological characteristics of the choroidal sublayer, according to the previously reported automatic analysis model of the choroidal sublayer [33], the choroid was divided into large, medium, and small vessel choroidal layers (LVCL, MVCL and SVCL, respectively), and the choroidal thickness (overall, LVCL, MVCL and SVCL) and vascular index (overall, LVCL and MVCL) were calculated. The measurement range is shown in Fig. 1.

There are 5 areas in total. The width of each area in the horizontal direction is 1 mm. The average choroidal thickness and CVI of each area were calculated. The middle position is the macular fovea. BM, Bruch’s membrane; CSI, choroidoscleral interface; SVCL-MVCL interface, interface of small-vessel choroidal layer and medium-vessel choroidal layer; MVCL-LVCL interface, interface of medium-vessel choroidal layer and large-vessel choroidal layer.

### Statistical analysis

The data were processed and analysed statistically using SPSS statistical software for Windows, version 23.0 (IBM Corp., Armonk, NY, USA). All values are presented as the mean ± standard deviation (SD). Categorical variables were assessed using the chi-square test, and the independent sample t test was used to evaluate differences in continuous variables for normally distributed variables. A value of *P* < 0.05 was considered statistically significant.

## Results

### Demographic data of the subjects

Thirty-three eyes of 33 patients (22 females, 11 males) with full-thickness IMHs (3 eyes in stage 2, 11 eyes in stage 3 and 19 eyes in stage 4) and 44 eyes of 44 patients (25 females, 19 males) with iERM (1 eye in stage 2, 19 eyes in stage 3 and 24 eyes in stage 4) were examined. The mean age of IMH patients was 67.12 ± 6.38 years and that of iERM patients was 69.43 ± 7.84 years. The IOP of IMH patients was 17.70 ± 1.86 mmHg, while that of iERM patients was 17.07 ± 2.35 mmHg. The AL of the IMH patients was 23.61 ± 0.91 mm, while that of the iERM patients was 23.28 ± 0.90 mm. The demographic and clinical characteristics of the patients are summarized and compared in Table 1. The BCVA measurements were significantly different between the 2 groups (*P* < 0.001), and there was no statistically significant difference in age, percentage of women, IOP, or AL between the 2 groups.

**Table 1:** Clinical Characteristics of the Participants

Characteristics	IMH (*n* = 33)	iERM (*n* = 44)	*P*
Age, year	67.12 ± 6.38	69.43 ± 7.84	0.171*
Sex (male/female)	11/22	15/29	0.945†
Eyes (right/left)	13/20	19/25	0.739†
Log-MAR VA	1.012 ± 0.360	0.559 ± 0.284	0.000*
IOP	17.70 ± 1.86	17.07 ± 2.35	0.209*
Axial length, mm	23.61 ± 0.91	23.28 ± 0.90	0.119*

Data are expressed as the mean ± SD

*IMH* Idiopathic macular hole, *ERM* Epiretinal membrane, *VA* Visual acuity, *IOP* Intraocular pressure

^*^Independent sample t test

^†^Fisher’s exact test

### Macular choroidal thickness measurements

**Fig. 2:**
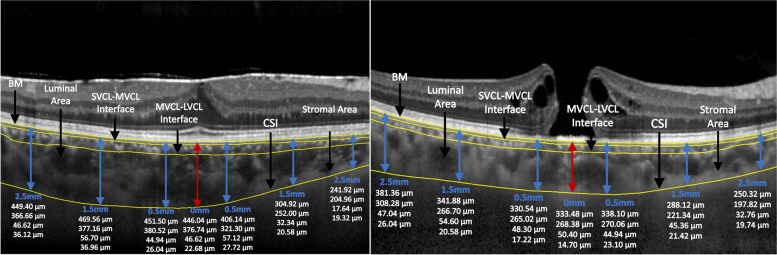
Representative figures of IMH and ERM

In the measured area, the central choroidal thickness (1 mm in the centre) in the IMH patients was almost the thinnest; in contrast, that of the iERM patients was the thickest, and the thickness generally decreased away from the fovea. The mean central choroidal thickness was 206.35 ± 81.72 µm in the IMH group and 273.33 ± 82.31 µm in the iERM group, with a statistically significant difference (*P* < 0.001). Table 2 shows the central choroidal thickness and the thickness of the nasal and temporal choroids 0.5–1.5 mm and 1.5–2.5 mm from the fovea of the two groups. The comparison of choroidal thickness between the two groups showed that the choroidal thickness of the central side, 0.5–1.5 mm, in the nasal and temporal sides in the IMH patients was significantly thinner than that in the iERM group (all *P* < 0.05). The choroidal thickness of 1.5–2.5 mm in the nasal and temporal sides in the IMH group was thinner than that in the iERM group, but there was no significant difference (*P* > 0.05). In further analysis of the thickness of the sublayers of the choroid, the thickness of the LVCL of the central choroidal thickness in the IMH group was thinner than that in the iERM group (*P* < 0.05), and there was no difference in the thickness of the other parts (*P* > 0.05). For the thickness of the MVCL, there was no difference from 1.5–2.5 mm on the temporal side, and the thickness of the other four parts in the IMH group was thinner than that in the iERM group (*P* < 0.05). The comparison of the SVCL thickness was consistent with that of the overall thickness. The representative figures of IMH and ERM are shown in Fig. 2.

**Table 2:** Choroidal Thickness in IMH and iERM Subjects

Choroidal Thickness	Location, mm From Fovea	IMH (*n* = 33)	ERM (*n* = 44)	*P*
	Temporal 1.5–2.5 mm	238.57 ± 49.16	245.30 ± 58.00	0.592
	Temporal 0.5–1.5 mm	230.65 ± 65.91	269.52 ± 73.00	0.018
OVERALL	Central 1 mm	206.35 ± 81.72	273.33 ± 82.31	0.000
	Nasal 0.5–1.5 mm	225.79 ± 65.72	259.20 ± 73.87	0.043
	Nasal 1.5–2.5 mm	205.92 ± 52.52	227.87 ± 60.17	0.099
	Temporal 1.5–2.5 mm	152.02 ± 35.35	153.61 ± 41.57	0.860
	Temporal 0.5–1.5 mm	153.90 ± 53.83	176.64 ± 55.67	0.076
LVCL	Central 1 mm	149.89 ± 58.37	185.11 ± 59.77	0.012
	Nasal 0.5–1.5 mm	151.25 ± 51.33	168.73 ± 52.25	0.147
	Nasal 1.5–2.5 mm	145.75 ± 56.19	143.01 ± 49.51	0.260
	Temporal 1.5–2.5 mm	64.04 ± 15.27	68.59 ± 25.22	0.362
	Temporal 0.5–1.5 mm	58.74 ± 17.06	69.42 ± 26.30	0.046
MVCL	Central 1 mm	47.62 ± 27.73	67.79 ± 29.75	0.003
	Nasal 0.5–1.5 mm	55.75 ± 17.19	68.05 ± 27.72	0.028
	Nasal 1.5–2.5 mm	54.14 ± 11.31	64.02 ± 16.17	0.004
	Temporal 1.5–2.5 mm	22.50 ± 1.95	23.09 ± 3.44	0.382
	Temporal 0.5–1.5 mm	18.02 ± 4.93	23.46 ± 4.64	0.000
SVCL	Central 1 mm	8.84 ± 6.72	20.43 ± 5.86	0.000
	Nasal 0.5–1.5 mm	18.78 ± 3.88	22.42 ± 5.27	0.001
	Nasal 1.5–2.5 mm	20.99 ± 1.84	20.84 ± 2.86	0.793

Data are expressed as the mean ± SD

Independent sample t test

*IMH* Idiopathic macular hole, *ERM* Epiretinal membrane, *LVCL* Large vessel choroidal layers, *MVCL* Medium vessel choroidal layers, *SVCL* Small vessel choroidal layers

The choroidal thickness in the macular centre of the IMH eyes was thinner than that of the iERM eye. From top to bottom, each column of data (white) represents the overall choroidal thickness, thickness of the LVCL, thickness of the MVCL, and thickness of the SVCL. BM, Bruch’s membrane; CSI, choroidoscleral interface; LVCL, large-vessel choroidal layer; MVCL, medium-vessel choroidal layer; SVCL, small-vessel choroidal layer.

### Macular CVI measurements

The mean CVI of the central choroid was 0.2480 ± 0.0536 in the IMH group, which was significantly higher than that in the iERM group (0.2120 ± 0.0616) (*P* < 0.05). The comparison of the CVI in other parts of the macula and the CVI in the LVCL and MVCL between the two groups showed no significant difference (all *P* > 0.05) (Table 3). The CVI of SVCL could not be detected.

**Table 3:** CVI in IMH and iERM Subjects

Choroidal CVI	Location, mm From Fovea	IMH (*n* = 33)	iERM (*n* = 44)	*P*
	Temporal 1.5–2.5 mm	0.1891 ± 0.0456	0.1772 ± 0.0506	0.292
	Temporal 0.5–1.5 mm	0.2000 ± 0.0679	0.1935 ± .0.0703	0.687
OVERALL	Central 1 mm	0.2480 ± 0.0536	0.2120 ± 0.0616	0.010
	Nasal 0.5–1.5 mm	0.1993 ± 0.0540	0.1775 ± 0.0788	0.181
	Nasal 1.5–2.5 mm	0.1875 ± 0.0556	0.1704 ± 0.0875	0.327
	Temporal 1.5–2.5 mm	0.2088 ± 0.0743	0.1952 ± 0.0767	0.438
	Temporal 0.5–1.5 mm	0.2062 ± 0.1017	0.2206 ± 0.0970	0.535
LVCL	Central 1 mm	0.2629 ± 0.0678	0.2376 ± 0.0852	0.175
	Nasal 0.5–1.5 mm	0.2158 ± 0.0796	0.2007 ± 0.1037	0.495
	Nasal 1.5–2.5 mm	0.1948 ± 0.0907	0.1823 ± 0.1285	0.636
	Temporal 1.5–2.5 mm	0.1721 ± 0.0567	0.1670 ± 0.0574	0.703
	Temporal 0.5–1.5 mm	0.2050 ± 0.0883	0.1708 ± 0.0670	0.070
MVCL	Central 1 mm	0.2186 ± 0.0629	0.1855 ± 0.0755	0.086
	Nasal 0.5–1.5 mm	0.2042 ± 0.0520	0.1767 ± 0.0750	0.100
	Nasal 1.5–2.5 mm	0.2139 ± 0.0715	0.1889 ± 0.0852	0.177

Data are expressed as the mean ± SD

Independent sample t test

*IMH* Idiopathic macular hole, *ERM* Epiretinal membrane, *LVCL* Large vessel choroidal layers, *MVCL* Medium vessel choroidal layers

## Discussion

The pathogeneses of IMH and iERM are closely related to the relationship between the vitreous and the macula. The difference between them is whether there is an abnormal adhesion in the vitreous macular region when posterior vitreous detachment occurs. The local pathological changes in the macula may be closely related to the exclusive occurrence of an abnormal adhesion in the macula. Changes in the choroid are likely to be related to local pathological changes in the macula. Previous studies have shown that chorioretinopathy involving the macula associated with choroidal thickening includes central serous chorioretinopathy, polypoid chorioretinopathy and Vogt Koyanagi Harada [34–36], while age-related macular degeneration, diabetic retinopathy, pathological myopia, and retinal dystrophy lead to a decrease in choroidal thickness [37–41].

The choroidal thickness measurement is affected by many factors, such as imaging time, refractive error, age, axial length, and sex. In this study, there was no difference in age or sex composition between the IMH and iERM groups. Axial length in the iERM group was shorter than that in the IMH group, but there was no significant difference. Therefore, the factors affecting choroidal thickness in the two groups were comparable. Most previous studies on idiopathic macular hole and choroidal thickness showed that the choroidal thickness was thinner than that of normal people. Although some studies did not correct the influencing factors of the choroid, such as axis length [24, 25], the same conclusion was reached in some studies that corrected axis length [4, 26]. The relationship between the iERM and choroidal thickness has not been determined. Some studies have found that the thickness of the iERM with traction is greater than that of the normal control group [30], but many studies have not found a relationship between the iERM and choroidal thickness [28, 29, 31]. Our results show that choroidal thickness in patients with IMH is lower than that in patients with ERM, with comparable ages and axial lengths, which is consistent with previous results.

Studies have shown that, although the choroidal thickness in the macular area of an IMH is thinner, the central thickness of the macula is still the thickest in the macular area [4, 24], and choroidal thickness was measured manually, rather than automatically calculated via the software instrument. Our study found that the choroidal thickness of 1 mm around the macular fovea is almost the thinnest in the macular area. Since our study measured the average thickness of 1 mm under the macular fovea of patients undergoing vitrectomy with an automatic analysis model, such inconsistency may be related to the method of measurement used and the composition of IMH patients.

The choroidal measurement method used in this study is an automatic recognition system based on deep learning choroidal sublayer thickness and CVI, which can measure choroidal thickness in different parts of the macula and choroidal thickness in different vascular layers [33] The results show that the choroidal thickness within 1.5 mm around the fovea in the IMH group is thinner than that in the iERM group, but there is no difference in the choroidal thickness 1.5–2.5 mm away from the fovea between the two groups, The choroidal thickness in the sublayers was analysed and showed that the thinning of the LVCL occurred within 1 mm of the fovea in the centre of the macula in the IMH group, and the thinning of MVCL and SMCL were basically consistent with the thinning of the whole choroid. These results showed that, compared with iERM, choroidal thinning of IMH mainly occurred in the MVCL and SVCL in the centre of the macula.

We also compared the CVI in the macular region between the two groups. The results showed that the CVI in the IMH group was higher than that in the iERM group within 1 mm of the macular fovea. The analysis of other parts and LVCL and MVCL showed that there was no difference in the CVI between the two groups. These results suggest that the increase in the CVI in IMH patients may mainly occur in the SVCL in the centre of the macula, which is roughly consistent with the thinning of choroidal thickness. We analysed the possible reasons why the CVI in the IMH group was higher than that in the iERM group. One is that the thinning of foveal choroidal thickness may mainly occur in the matrix, and the other is the compensatory change in the choroidal microvessels after the thinning of foveal choroidal thickness.

The results of this study show that after excluding influencing factors such as age, sex and axial length, the choroidal thickness of IMH is significantly lower than that of iERM, indicating that the difference in choroidal thickness is an independent influencing factor between these two vitreo-macular interface disorders. Our study and most previous studies are cross-sectional, so a causal relationship could not be determined. It has been found that changes in choroidal blood perfusion occur before the complete formation of macular holes [42]. Some authors also conducted a prospective study on patients with macular thinning and found that 86% (6/7) of patients with macular thinning, pigment epithelial window defects and no PVD developed a macular hole [43]. These results show that the structural changes in the macula are closely related to the occurrence of IMH.

In view of the above results, we found that changes in choroidal thickness play an important role in the pathogenesis of IMH and iERM. First, the normal choroidal thickness in patients with iERM indicates that the blood and oxygen supply of the choroid is basically normal. Its effect on the vitreous body may be due to the ageing of the vitreous body caused by the relatively normal metabolism of the choroidal retina and the occurrence of PVD, which may lead to the formation of the iERM. Second, macular choroidal thickness decreased in patients with IMHs. Although there may be compensatory changes in choroidal vessels, they may still affect the metabolism of the macula. The local metabolic abnormality of the macula may lead to the adhesion of the vitreous macula, resulting in a macular hole. Another disease of macular choroidal thickness reduction and abnormal adhesion of the vitreoretinal region is high myopia. The macular complication of high myopia is also due to the adhesion and traction of the vitreous to the macula, resulting in retinoschisis and a macular hole [44, 45].

There are a few limitations in our study. First, this group of cases is a retrospective study of patients who underwent vitrectomy. Most patients are in the later stage of the disease, which may lead to selection bias. Second, there were relatively few cases in this group, which were not compared with the normal control group and need to be supplemented by follow-up research. Thirdly, functional parts of the choroid can be evaluated by using ICGA and OCTA; however, these are not routine examinations in our hospital. Therefore, we did not conduct any such analyses.

In summary, we concluded that the choroidal thickness of an IMH was significantly thinner than that of the ERM, which was mainly manifested in 3 mm in the macular centre and the MVCL and SVCL layers of the choroid after excluding influencing factors such as age, sex, and axial length. The choroidal vascular index of an IMH was higher than that of the iERM. These findings suggest that the choroid may be involved in the pathogenesis of IMH and iERM.

## Data Availability

The datasets used and/or analyzed during the current study are available from the corresponding author on reasonable request.
